# Determination of the myocardial area at risk after reperfused acute myocardial infarction with different imaging techniques: cardiac magnetic resonance imaging, multidetector computed tomography and histopathological validation

**DOI:** 10.1186/1532-429X-13-S1-O9

**Published:** 2011-02-02

**Authors:** Nathan Mewton, Stanislas Rapacchi, Lionel Augeul, René Ferrera, Joseph Loufouat, Loic Boussel, Gilles Rioufol, Didier Revel, Michel Ovize, Pierre Croisille

**Affiliations:** 1 Hôpital Cardiovasculaire Louis Pradel, LYON, France; 2 CREATIS-LRMN, CNRS UMR 5220 – INSERM U630 – Université Claude Bernard Lyon 1, Lyon, France; 3 Inserm U886 Cardioprotection, Université Claude Bernard Lyon1, Lyon, France; 4 Hôpital Cardiovasculaire Louis Pradel/ CREATIS-LRMN, CNRS UMR 5220 – INSERM U630 – Université Claude Bernard Lyon 1, Lyon, France

## Introduction

The myocardial area at risk (AAR) is a major determinant of infarct size. Which imaging technique is the most appropriate to accurately measure its size remains debated.

## Purpose

The principal objective of this study was to compare the AAR defined with two different T2 weighted cardiac magnetic resonance (T2W CMR) imaging sequences (TIRM T2w blood suppressed TSE and ACUTE TSE-SSFP), the contrast-enhanced (ce-) CMR endocardial surface length (ESL) after 90-minutes of reperfusion and the arterial enhanced multi-detector computed tomography (MDCT) performed during occlusion with the reference histological AAR delineated after injection of uniperse blue dye in reperfused myocardial infarction.

## Methods

**Figure 1:**
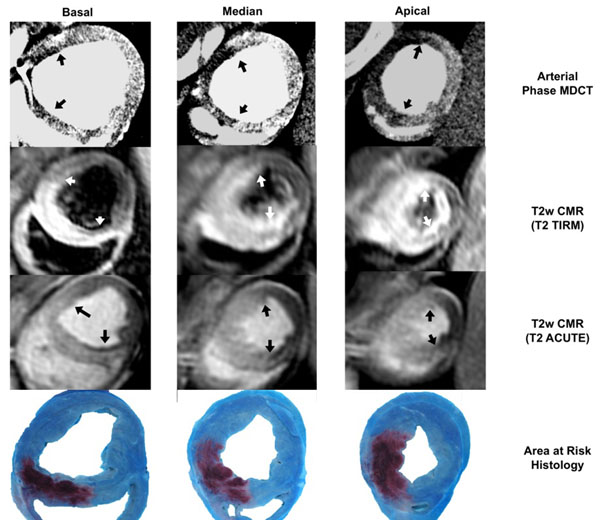


**Figure 2:**
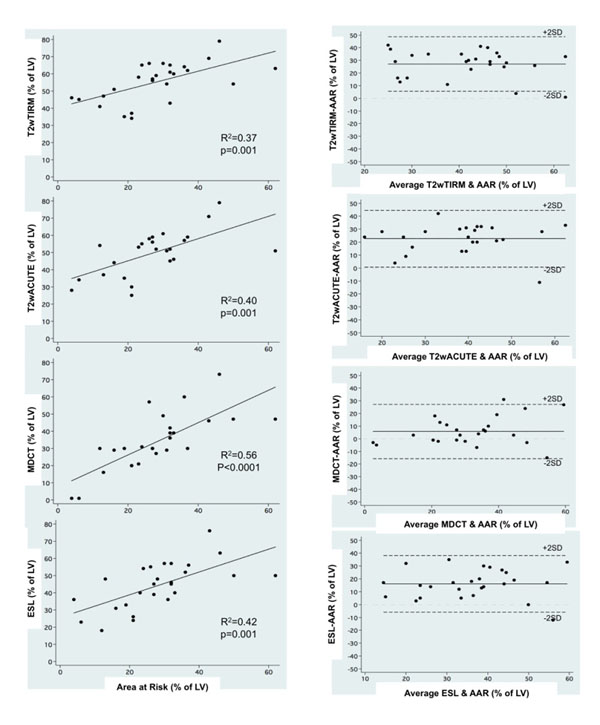


Fifteen closed-chest pigs underwent a 40-minutes coronary artery occlusion (angioplasty balloon inflation), followed by reperfusion. Three co-registered short-axis slices (base, mid-ventricle, apex) were obtained for each animal and each imaging technique for statistical analysis (Figures 1 and 2).

## Results

The best fit with the reference histological AAR was obtained for the hypoenhanced area on arterial enhanced MDCT (R2=0.56; P<0.05) with a small bias on Bland-Altman plots (5.7±11% LV area). The AAR as defined by both T2W TIRM and ACUTE sequences or the ESL on ce-CMR significantly overestimated the size of the AAR by pathology with only a fair correlation (R2=0.37, R2=0.40 and R2=0.42; P<0.05 respectively) and important bias (27.2 ± 11.0% LV area; 22.6 ± 11.2% LV area 16.0±11.3% respectively).

## Conclusions

Arterial enhanced MDCT performed at the time of occlusion was the most accurate method to assess the AAR, whereas T2wCMR and the contrast enhanced ESL performed 90 minutes after reperfusion significantly overestimated the AAR.

